# A Case of Acute Mechanical Mitral Valve Thrombosis Management With Venoarterial Extracorporeal Membrane Oxygenation

**DOI:** 10.7759/cureus.55944

**Published:** 2024-03-11

**Authors:** Joana Alves Cabrita, Cleide Barrigoto, Raquel Maia, Maria João Oliveira, Philip Fortuna

**Affiliations:** 1 Intensive Care Unit, Centro Hospitalar Universitário de Lisboa Central, Lisbon, PRT; 2 Intensive Care Unit, Hospital Prof. Doutor Fernando Fonseca, Lisbon, PRT

**Keywords:** case report, cardiogenic shock, va-ecmo, prosthetic valve thrombosis, mechanical mitral valve complications, mitral valve surgery

## Abstract

Mechanical prosthetic valve thrombosis (PVT) and obstruction are rare and dangerous events often related to inappropriate anticoagulant therapy. High mortality rates occur because of delayed diagnosis, hemodynamic instability, multiple organ failure (MOF), and high perioperative risk. Surgical repair is a first-line treatment for obstructive PVT with hemodynamic instability but is often not readily available or safely performed. Venoarterial extracorporeal membrane oxygenation (VA ECMO) support has been increasingly used in patients with PVT and cardiorespiratory collapse, allowing MOF reversal and safer deferred surgery. The authors present a case of a young female with refractory cardiogenic shock secondary to mitral PVT successfully managed with VA ECMO. Furthermore, the promising role of perioperative VA ECMO support for PVT-related cardiogenic shock is also discussed.

## Introduction

Prosthetic valve thrombosis (PVT) refers to the formation of blood clots on mechanical heart valves, which can lead to valve dysfunction and potentially life-threatening complications, such as thromboembolism, valve obstruction, and cardiogenic shock [1]. Although more thrombogenic, mechanical heart valves have higher durability, with an annual rate of PVT of 0.1-5.7% in developed countries (while the incidence in developing nations is higher). The main mechanisms involved in PVT include hemodynamic factors (stasis and turbulence), complement activation, platelet aggregation, and hypercoagulability states [1].

Mitral valve PVT is more common (two to three times) than aortic valve thrombosis [2,3]. Anticoagulant therapy, such as vitamin K antagonists, is routinely prescribed to patients with mechanical heart valves to prevent blood clots and reduce the risk of PVT. However, inadequate anticoagulation remains the most frequent cause of valve thrombosis [2,3].

Clinical presentation is heterogeneous and is dictated by valve obstruction, which, although rare (incidence of 0.3-1.3% patient-years [4]), usually evolves into cardiogenic shock and multiple organ failure (MOF) because of complete blood flow interruption. There is also an increased risk of systemic embolism, especially with concurrent endocarditis. Nonobstructive PVT is usually an indolent process. Systemic embolization can still be present, but the disease is typically asymptomatic, and the diagnosis is frequently made during routine echocardiography [4].

Diagnosis is made by transthoracic echocardiography (TTE), transesophageal echocardiography (TEE), cinefluoroscopy, or cardiac CT (CCT) [5]. TTE usually shows a high gradient or velocity across the prosthesis (with or without the presence of mobile densities) and reduced leaflet motion [2]. A mean gradient >8 mmHg and an effective area ≤1.3 cm^2^ indicates PVT in the case of a mitral prosthesis [3,4].

Management of mechanical PVT is hazardous and has a mortality of 10%, independent of treatment choice [4]. For left-sided PVT, emergency surgery and continuous infusion of a low-dose thrombolytic therapy are both effective options. The choice is based on clinical factors and local expertise [2,4,6]. Slow infusion of thrombolytic therapy can achieve hemodynamic success rates >90%. However, if the patient has a prohibitive condition to fibrinolysis, the alternative is emergent surgery. Emergent valve replacement in critically ill patients with MOF has a very high perioperative risk: that risk is correlated with the level of circulatory impairment and New York Heart Association (NYHA) functional class on presentation (4% for patients with NYHA I, II, III; 17.5% in patients with NYHA class IV symptoms) [3,4].

Venoarterial extracorporeal membrane oxygenation (VA ECMO) is an extracorporeal support system that provides circulatory support during a limited time, allowing immediate cardiopulmonary support [7]. The main indication for VA ECMO is refractory cardiogenic shock, which includes refractory cardiac arrest in the worst-case scenario. It has been used as a bridge to recovery, bridge to heart transplant, or bridge to long-term mechanical support in the setting of acute coronary syndrome, myocarditis, refractory ventricular arrhythmias, hypothermia, post-cardiotomy, and primary graft failure after heart transplantation [1-2,7-14].

Because of retrograde blood flow against the aortic valve, in peripheral VA ECMO, the risk of intracavitary stasis and thrombosis is very high, so the technique requires systemic anticoagulation. VA ECMO is also associated with bleeding [15] because of systemic anticoagulation and activation of coagulation factors, nonpulsatile flow, and intracardiac stasis. The most common anticoagulation strategy is unfractionated heparin (UFH) [14], guided by anti-Xa or activated partial thromboplastin (APTT) targets. High risk of bleeding and severe thrombocytopenia are known contraindications for this extracorporeal support. Still, each case should be discussed and decided if the technique is performed without anticoagulation, which is usually avoided on VA ECMO. A systematic review [15] analyzed thrombotic and bleeding outcomes on ECMO and described an overall incidence of bleeding of 32.8% and thrombosis of 21.9%, with 8.5% of patient thrombosis mainly arterial on VA ECMO. Hemolysis induced by the ECMO technique is also commonly described, with an incidence from 5% to 18% [16]. It consists of the mechanical injury of red blood cells provoked by several factors that cause high shear stress [16]. Nonpulsatile blood flow provided by the machine, blood contact with an artificial ECMO circuit, and frequent transfusion requirements are conditions that induce hemolysis and endothelial activation. PVT by itself can also cause red blood cell disruption. The degree of hemolysis can vary, and there are no studies about the cumulative hemolysis risk in a patient with PVT and VA ECMO support.

Although VA ECMO application as a bridge to surgery in critically ill patients has risen in recent years [1,7,13-14], there is still scarce literature about VA ECMO indication in PVT. The risk of thrombosis, bleeding, and hemolysis is very high when VA ECMO is used as a bridge to surgery in the case of PVT. However, some case reports suggest this could be a good strategy for selected patients [3,8-11,13] treated in comprehensive/primary valve centers [2].

## Case presentation

A 19-year-old woman with previous rheumatic heart disease, secondary pulmonary hypertension, and tricuspid regurgitation presented to a general hospital with two-day symptoms of shortness of breath and hemoptysis. In the past, she was submitted to mitral and tricuspid valvuloplasty and a mechanical mitral valve prosthesis, being chronically treated with vitamin K antagonist (VKA). Difficulty with international normalized ratio (INR) control (previous weekly measures around two, below the target of 2.5-3.5) was described. The patient also stopped the VKA by self-initiative the day before admission because of hemoptysis.

The peripheral oxygen saturation at room air was 90% at admission, and nasal cannula oxygen was administered. COVID-19 infection was excluded, and blood tests (Table 1) showed low inflammatory parameters (15.7 x 10^9/L leucocytes, protein C-reactive of 2.26 mg/dL, procalcitonin of 0.05 ng/mL), D-dimers of 3121 ug/L, hemoglobin of 10.3 g/dL, and INR 1.2.

**Table 1:** Admission laboratory results INR: international normalized ratio. Admission laboratory results (with normal range and units).

Laboratory test	Normal range	Result
Leucocytes	4.0 x 10^9/L-10.0 x 10 ^9/L	15.7 x 10^9/L
Protein C-reactive	<0.5 mg/dL	2.26 mg/dL
Procalcitonin	<0.05 ng/L	0.05 ng/L
D-dimers	<230 ug/L	3121 ug/L
Hemoglobin	12-15 g/dL	10.3 g/dL
INR	0.8-1.2 (may differ depending on the target)	1.21

**Figure 1:**
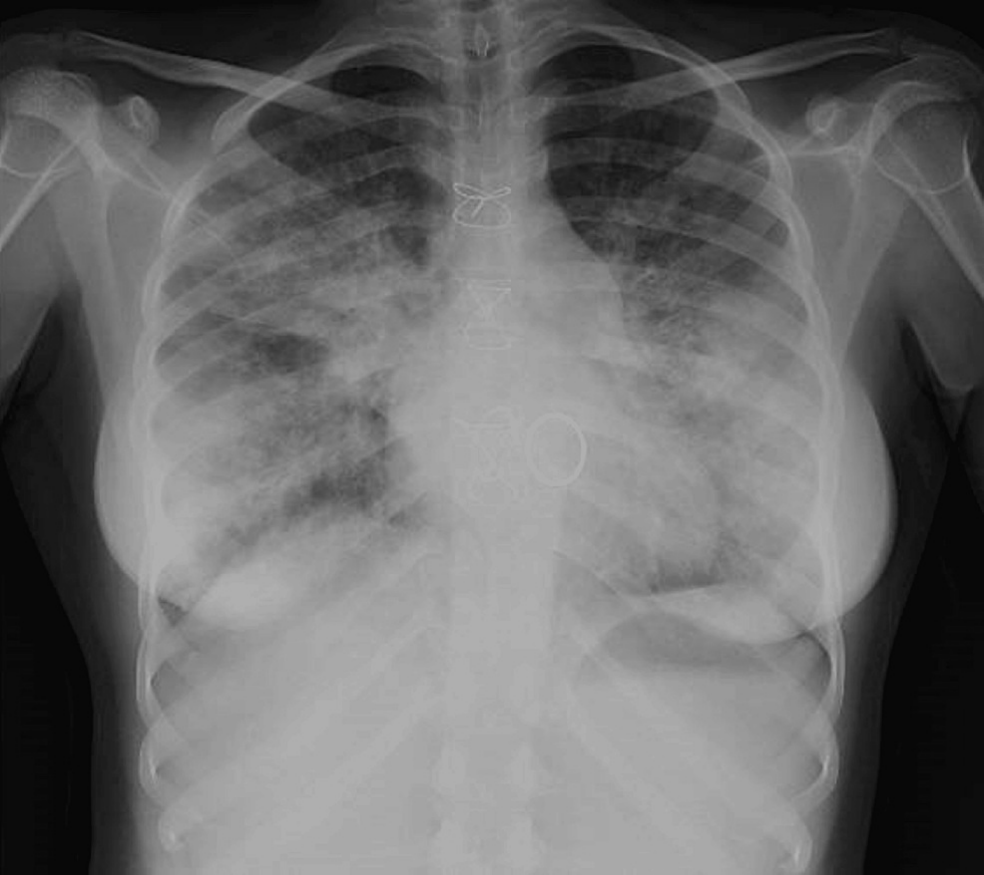
Thoracic X-ray on admission Thoracic X-ray with bilateral alveolar infiltrates.

**Figure 2:**
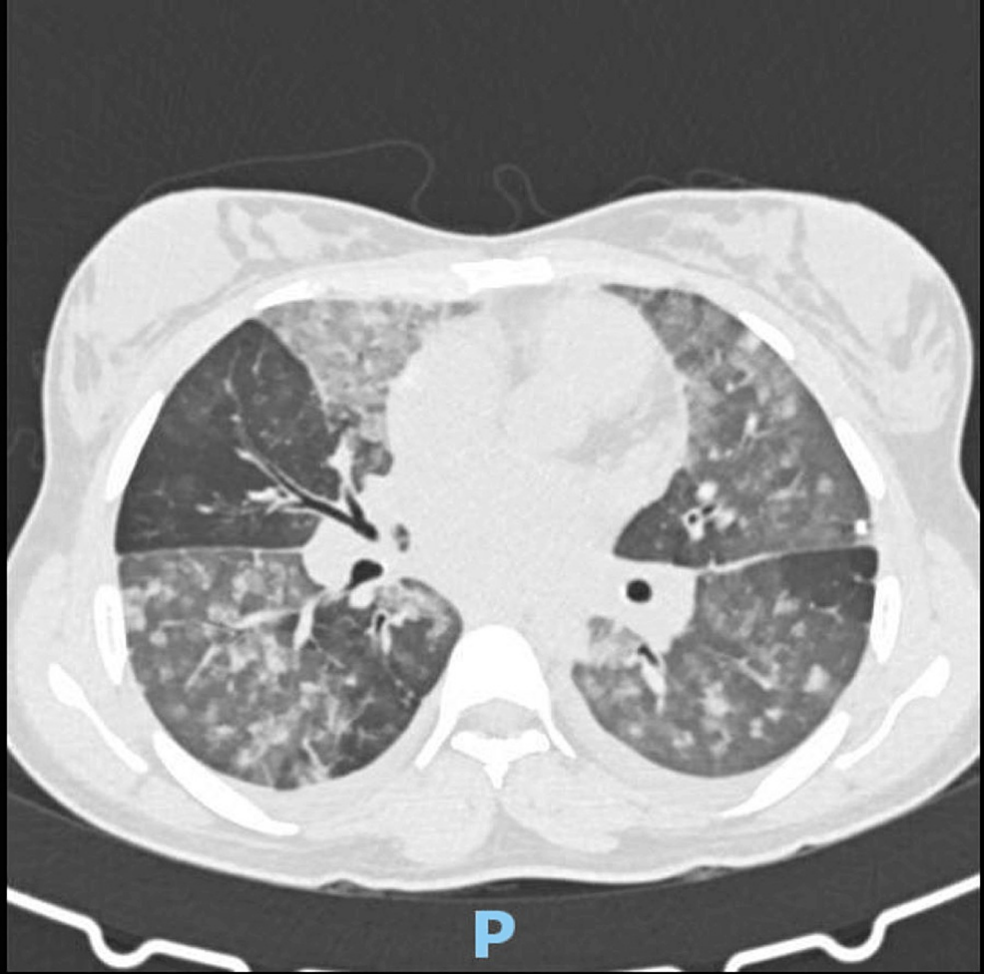
Thoracic CT scan on admission Thoracic CT scan showing extensive infiltrate in both lungs with areas of ground glass opacification and mild pleural effusions.

Thoracic X-ray revealed fully bilateral alveolar infiltrates (Figure 1), and thoracic CT scan showed an extensive infiltrate in both lungs with areas of ground glass opacification and mild pleural effusions (Figure 2).

She stayed in the emergency room for 24 hours, with persistent hemoptysis and a concomitant decrease in hemoglobin (10.3 g/dL to 6.9 g/dL), which is why she remained without anticoagulation. Respiratory failure got worse, and ventilatory support was gradually increased to a high-flow nasal cannula and non-invasive mechanical ventilation without improvement.

She was intubated and admitted to the intensive care unit (ICU), where a bronchoscopy was performed, and the clinical diagnosis of alveolar hemorrhage was established. As refractory respiratory insufficiency persisted (PaO2/FiO2 < 120), neuromuscular blocking and prone position ventilation were initiated. A hemodynamic instability was also observed (lactate of 9 mmol/L), and fluid and vasopressor support (norepinephrine) were initiated. A TTE was performed and revealed a severe right ventricle dilatation with bouncing of the septum into the left ventricle, pulmonary artery hypertension (pulmonary systolic artery pressure (PSAP) of 50 mmHg), and reduced movement of mitral valve leaflets. A TEE confirmed the presence of an acute thrombosis of the mitral valve with a mean transvalvular gradient of 24 mmHg and acute right heart failure. UFH perfusion was immediately started. Considering the presence of right ventricle dysfunction with acute respiratory failure, inhaled milrinone and nitric oxide were also begun until definitive solution treatment was arranged.

Despite the therapeutic measures established, the patient continued to deteriorate and progressed to severe metabolic acidosis and MOF. As thrombolytic therapy was not an option because of active alveolar bleeding, emergency surgery became mandatory, but the patient was too unstable to be safely transferred to a facility with cardiac surgery and too sick for immediate operative stress. After a multidisciplinary discussion between cardiac surgery, the ECMO team, and the responsible medical team, a decision was made to use VA ECMO as a bridge to cardiac surgery.

A mobile care ECMO team went to the local ICU and performed a percutaneous cannulation of the right femoral artery (17Fr, 23cm, Maquet GetingeⓇ; Maquet Getinge Group, Rastatt, Germany) and vein (23 Fr, 55cm, Maquet GetingeⓇ; Maquet Getinge Group, Rastatt, Germany) under echographic guidance. A distal perfusion cannula was also positioned in the common right femoral artery (CruraSaveⓇ, 8Fr; Vatska Medtech Private Limited, Chennai, India). Peripheral VA-ECMO was initiated, hemodynamic stability was restored, and the patient was transported to the closer ECMO center with cardiac surgery.

**Figure 3:**
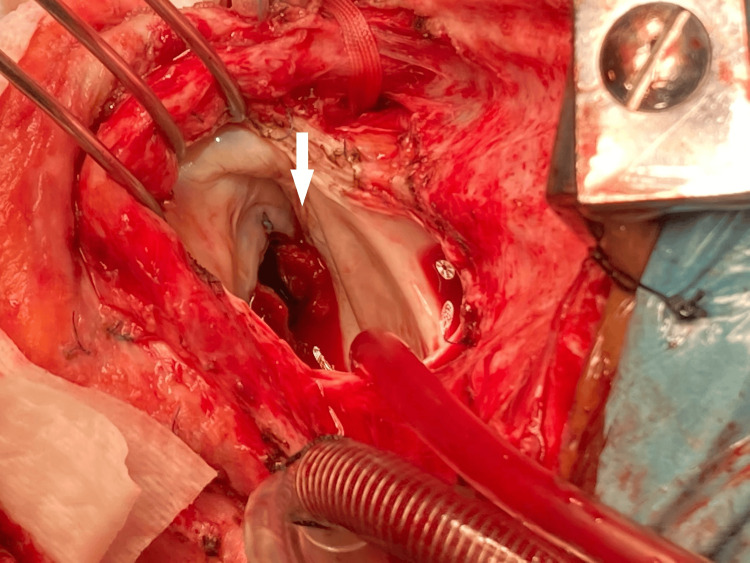
Prosthetic valve thrombosis of mitral valve PVT of mitral valve (auricular view) indicated by a white arrow.

**Figure 4:**
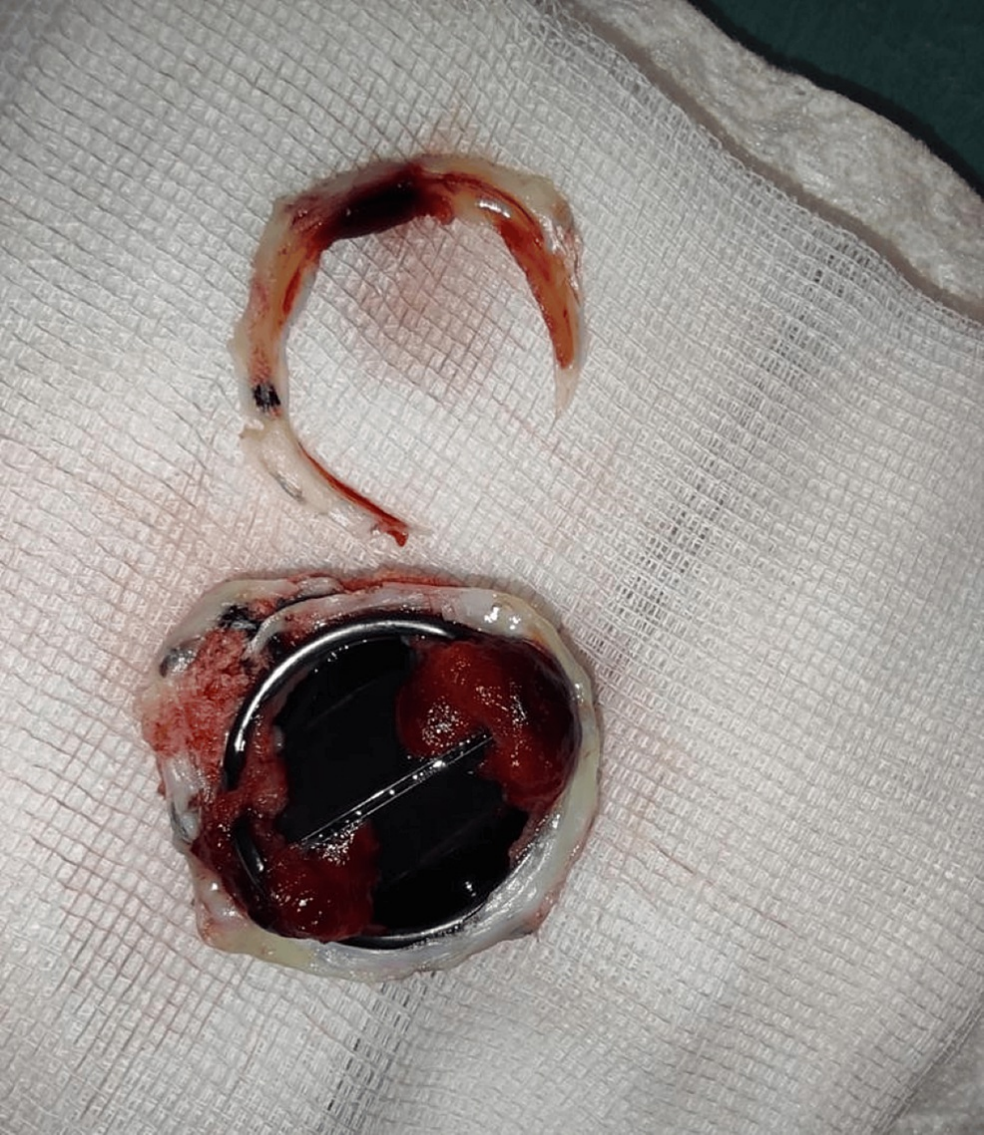
Explanted mitral valve prosthesis Explanted mitral valve prosthesis with thrombus (superior view).

VA ECMO support was continued for 72 hours, with UFH perfusion (targeted to anti-Xa levels of 0.3-0.5 IU/mL). During that time, the patient's hemodynamics and organ perfusion improved, allowing the weaning of inotropes and vasopressors. After three days of circulatory optimization, the patient recovered their renal and liver function and was taken to the operating room for mitral prosthetic valve replacement and decannulation from VA ECMO. A moderate thrombus was found in the prosthetic valve (Figures 3,4).

The thrombus was removed, and a new prosthetic valve replaced the prosthetic valve. The patient tolerated surgery well, came out of cardiopulmonary bypass without needing complementary circulatory support, was extubated on the first postoperative day, and weaned from all vasopressors.

She made a full recovery and was discharged on the fifteenth postoperative day.

Six months after discharge, the patient remained under VKA anticoagulation with an adequate INR. No additional symptoms nor limitations to her daily active life were reported.

## Discussion

The risk of PVT is related to endothelial, hemodynamic, and hemostatic factors and is higher with mechanical heart valves in the perioperative period and in association with subtherapeutic anticoagulation [1]. The obstruction of a right-sided mechanical prosthesis (particularly tricuspid) is more common than left-sided PVT because of hemodynamic factors, and mitral PVT is more frequent than aortic PVT [1-4].

Clinical presentation depends on whether an obstruction is present or not. Usually, a severe valve obstruction presents with apparent heart failure [4]. Management of mechanical PVT is a demanding process. Fibrinolysis is associated with an increased risk of bleeding, embolization, and recurrent venous thromboembolism. VA ECMO has been used to provide cardiorespiratory support in collapsed patients who failed conventional management [3,7,8]. However, this technique may be associated with bleeding complications and with thrombotic events [14]: overall incidence of 21.9% of thrombosis on ECMO, wherein 8.5% is in the arteries of VA ECMO patients [15].

A patient with a known PVT constitutes a setting where VA ECMO dynamics (primarily because of increased left ventricular afterload) can increase the risk of intracavitary stasis even more. However, the dismal outcomes in critically ill patients in need of emergent surgery have led to a new approach where emergent cardiopulmonary support is used to provide stabilization to a postponed intervention [12]. Although already proven by small cohorts, there is still scant evidence to guide patient selection. A multidisciplinary discussion is essential to avoid futility and improve survival. To our knowledge, only a few case reports wherein VA ECMO was used as a bridge to valve replacement surgery in acute PVT, and only three were related to mechanical mitral valve PVT [3,10,13].

In this case, the patient suffered an acute obstructive thrombosis of a mechanical mitral valve. Besides the presence of hemoptysis and alveolar hemorrhage, which may be associated with mitral acute disease and anticoagulation, the symptoms at admission were nonspecific and quickly progressed to respiratory failure and cardiogenic shock with MOF. Mitral disease (both stenosis and regurgitation) can lead to pulmonary congestion and hemoptysis because of increased pressures (with high left atrial pressure) that induce the rupture of pulmonary vessels. Diffuse alveolar hemorrhage is a rare presentation, but it is described in the setting of acute cardiogenic pulmonary edema [17].

The thrombotic event was probably associated with inadequate anticoagulation as the patient stopped VKA therapy, and previously, INR control was scarce: the INR at admission was highly below the recommended range for mechanical valves (2.5-3.5) [5]. Considering that she was admitted to a hospital without cardiac surgery and thrombolytic therapy was not an option because of hemoptysis, emergent mitral valve replacement and transfer to another facility were mandatory. VA ECMO ensured complete and immediate cardiopulmonary support, which facilitated the safe transfer to an ECMO center with cardiac surgery facilities. Immediate valve replacement was considered dangerous in a patient with MOF. Balancing the thrombotic/bleeding risks and possible benefits, the team decided to keep the patient on VA ECMO for 72 hours to improve the patient's hemodynamics and maintain adequate organ perfusion. As the patient was under UFH before VA ECMO initiation and the thrombotic risk was not negligible, the ECMO team decided to keep systemic UFH as usually recommended for the technique. After stabilization, definitive surgery was performed, and the patient was decannulated. No other organ support measures were needed.

A particular point to note in this case is the feasibility and availability of VA ECMO in resource-constraint settings, regarding that ECMO support carries high costs per individual patient, and its availability has significant variation worldwide.

## Conclusions

Although rarely used for this purpose, VA ECMO can be a viable option for patients with obstructive PVT that present in refractory cardiogenic shock. Although the balance between thrombotic risk and bleeding complications remains challenging, technology improvements and management by experienced teams have made VA ECMO simpler and safer. In highly unpredictable perioperative settings, as described in our case report, a two-way strategy with VA ECMO stabilization followed by deferred surgery may improve prognosis and reduce mortality. However, in each situation, the benefits and potential risks should be carefully analyzed to make a safe decision.
